# Outcomes of Laparoscopic Versus Open Lateral Lymph Node Dissection Following Total Mesorectal Excision for Advanced Rectal Cancer: A Systematic Review and Meta-Analysis

**DOI:** 10.7759/cureus.107358

**Published:** 2026-04-19

**Authors:** Rubens Rigo, Luis Alves, Vinnicius Duarte, Pedro Franco, Bernardo F Pompeu, Fernanda Formiga

**Affiliations:** 1 Urology, Hospital Universitario Evangelico Mackenzie, Curitiba, BRA; 2 General Surgery, Heliopolis Hospital, São Paulo, BRA; 3 Colorectal Surgery, Hospital Heliopolis, São Paulo, BRA; 4 Colorectal Surgery, Santa Casa de São Paulo, São Paulo, BRA

**Keywords:** colorectal cancer, lateral pelvic lymph node dissection, open and laparoscopic surgery, systematic review, total mesorectal excision

## Abstract

This study aims to compare laparoscopic vs open lateral pelvic lymph node dissection (LPLND) for locally advanced rectal cancer to assess operative outcomes and postoperative complications. A systematic search was conducted in PubMed, Scopus, and the Cochrane Central Register of Clinical Trials for studies published up to June 2025. Odds ratios (ORs) and mean differences (MDs) with 95% confidence intervals (CIs) were pooled using a random-effects model. Heterogeneity was assessed using I² statistics, and statistical analyses were performed in R Software version 4.4.1 (R Foundation for Statistical Computing, Vienna, Austria). Eight retrospective trials involved 896 patients undergoing total mesorectal excision plus LPLND for treatment of rectal cancer, of whom 470 (52.4%) underwent the laparoscopic and 426 (47.6%) open surgery. The laparoscopic group showed lower intraoperative blood loss (MD = −732.8 ml; 95% CI: −1328.1 to −83.5; p = 0.03; I² = 95%) and lower need for blood transfusion (OR = 0.28; 95% CI: 0.16 to 0.49; p < 0.0001; I² = 6%). Trends favoring the laparoscopic group were observed for overall postoperative complications (OR = 0.65; 95% CI: 0.41 to 1.01; p = 0.057; I² = 50%), and wound infection (OR = 0.54; 95% CI: 0.28 to 1.03; p = 0.063; I² = 0%). No statistically significant differences were observed between groups for abdominal abscess, intestinal obstruction, Clavien-Dindo complications grade ≥ 3, lower limb neuropathy, anastomotic and lymphatic leakage, total lymph nodes harvested, LPLN harvested, operative time, postoperative hospital stay, and time to a soft diet. In conclusion, laparoscopic LPLND has been associated with reduced intraoperative blood loss and seems to confer improved safety and effectiveness in the treatment of rectal cancer.

## Introduction and background

Colorectal cancer is the third most common cancer worldwide and the second leading cause of cancer-related death globally [1]. In 2020, there were 1.93 million new cases and nearly 1 million deaths, with projections indicating an increase to 3.15 million new cases and 1.6 million deaths by 2040, especially in developing countries [2]. Approximately 25% of cases involve the rectum, and among these, 10 to 20% present with concomitant metastasis to the lateral pelvic lymph node (LPLN) chain, which increases the risk of recurrence and is associated with a worse prognosis [1,3]. Treatment strategies differ between Western and Eastern countries. In the West, LPLN metastasis is considered a systemic disease, and LPLN dissection (LPLND) is associated with longer operative time and increased perioperative complications. Therefore, total mesorectal excision (TME) combined with chemoradiotherapy (CRT) is preferred for lymph node metastases [4]. In the East, however, TME with CRT alone is considered insufficient, as these cases are classified as locally advanced disease, and the addition of lateral lymph node dissection (LLND) has been shown to reduce the risk of recurrence by up to 10% [5].

Despite the clear advantages of robotic surgery, this approach is not equally accessible in developing countries, and many surgeons must choose between laparoscopic and open surgery. The previous meta-analysis by Du et al. included 1,176 patients; however, it did not incorporate propensity score-matched analyses from the included studies, had potential population overlap, and considered robotic-assisted procedures [6]. Since 2020, new studies have been published comparing laparoscopic and open surgery, adding over 500 patients to the literature, which justifies the need to update this meta-analysis.

## Review

Methods

This systematic review followed the Preferred Reporting Items for Systematic Reviews and Meta-Analysis (PRISMA) guidelines [7]. The study protocol was registered with the International Prospective Register of Systematic Reviews (PROSPERO) under the registration number CRD420251068334 [8].

Search Strategy

A systematic search was performed across major bibliographic databases for studies published up to June 2025. The search strategy was as follows: ("Colorectal Neoplasms" OR rectal cancer OR rectal neoplasm* OR cancer of the rectum OR rectum cancer OR rectal carcinoma OR rectal tumor* OR rectal malignan*) AND ("Lymph Node Excision" OR pelvic lymphadenectomy OR lateral lymph node dissection OR lateral pelvic lymphadenectomy OR lateral pelvic lymph node OR lateral pelvic wall lymph-node dissection OR extended lymphadenectomy OR pelvic side wall dissection OR lateral lymphadenectomy OR pelvic lymph node dissection OR LLND) AND ("Laparoscopy" OR laparoscop* OR minimally invasive surgery OR laparoscopic surgery).

Eligibility Criteria

We included non-randomized controlled trials (n-RCTs) that compared laparoscopic LLND with open LLND in adult patients undergoing TME for the treatment of rectal cancer. The exclusion criteria were as follows: (1) robotic approach; (2) single-arm studies; (3) publications deemed ineligible for inclusion, such as case reports, conference abstracts, meta-analyses, reviews, and animal studies; and (4) studies with overlapping patient populations.

Data Extraction and Endpoints

Two authors (R.S.M.R. and V.N.T.D.) independently selected articles that met the inclusion criteria using the Rayyan app and extracted data from the selected studies. After that, the extracted data were placed in an Excel spreadsheet (Microsoft Corp., Redmond, WA, USA). Any disagreements were resolved by consensus or, if necessary, by consulting a third author (B.F.P.). The primary outcomes were defined as indicators of safety and feasibility, including intraoperative blood loss, need for transfusion, mortality, and overall postoperative complications. The secondary endpoints comprised other perioperative complications, such as anastomotic leakage, conversion rate, intestinal obstruction, abdominal abscess, Clavien-Dindo grade ≥ III complications, lymphatic leakage, neuropathy in the lower limb, wound infection, operative time, postoperative hospital stay, time to soft diet, total lymph nodes harvested, and LPLN harvested.

Quality Assessment

Two authors (R.S.M.R. and P.P.F.) independently assessed the quality of included studies using the Risk of Bias in Non-randomized Studies of Interventions (ROBINS-I) [9]. In this assessment, each study is categorized as low, moderate, serious, or critical risk across seven domains: confounding, participant selection, classification, deviations from intended interventions, missing data, outcome measurement, and selection of the reported result. Disagreements were resolved unanimously through interventions with the senior author (B.F.P.).

Statistical Analysis

We pooled odds ratios (ORs) for binary outcomes and mean differences (MDs) for continuous endpoints, with 95% confidence intervals (CIs). All pooled estimates were generated using a random-effects model with restricted maximum likelihood. Statistical significance was defined as p < 0.05. Heterogeneity was assessed using the Cochran Q test and I² statistic, with p-values < 0.10 and I² > 25% considered significant. For outcomes with substantial heterogeneity, we used Baujat plots to assess each study’s contribution to the overall effect and heterogeneity. Furthermore, we also performed leave-one-out sensitivity analyses by systematically removing each study from the pooled estimates to ensure the robustness of the results. R Software version 4.4.1 (R Foundation for Statistical Computing, Vienna, Austria) was used for statistical analysis.

Results

Study Selection and Characteristics

**Figure 1:**
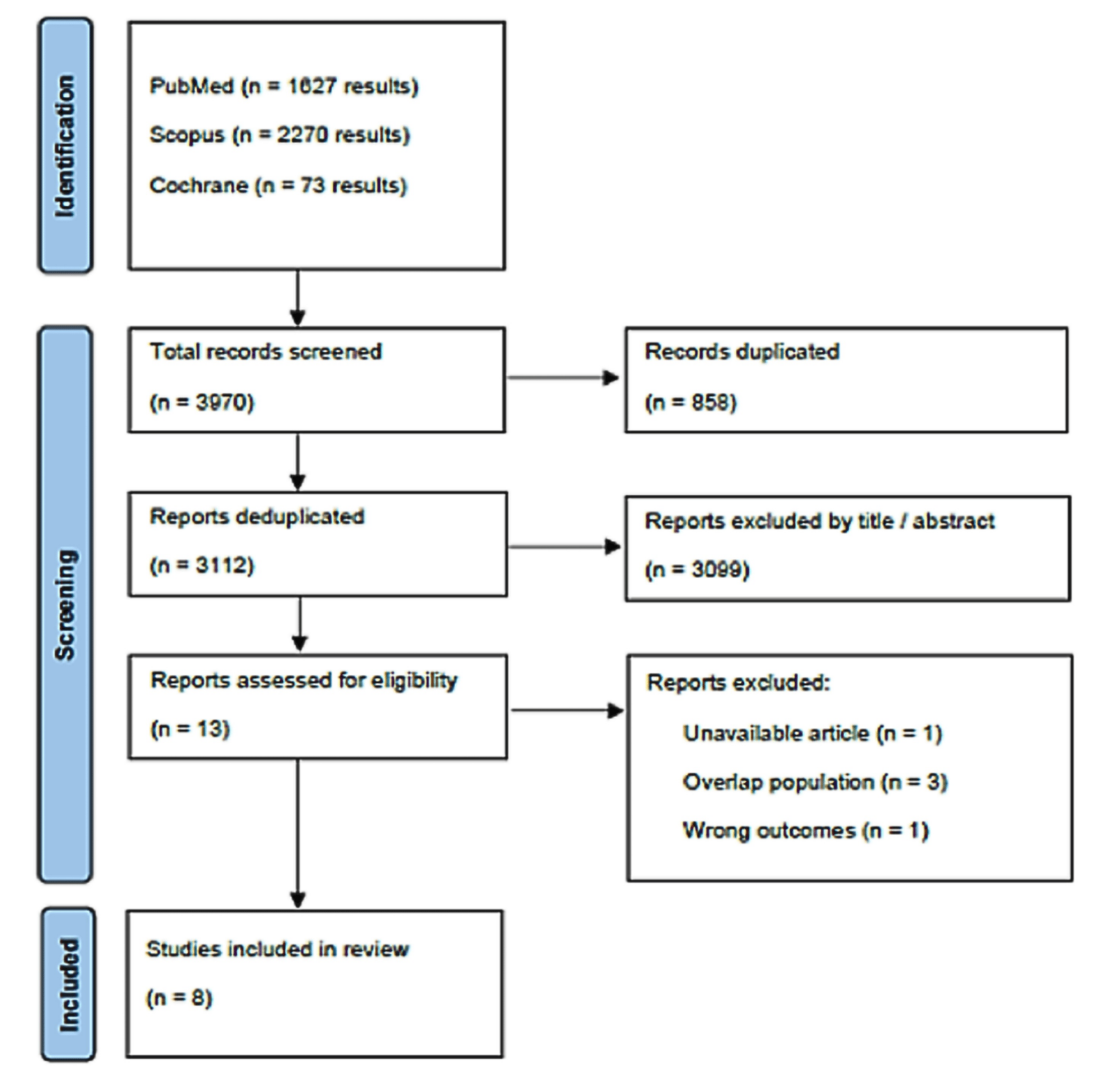
PRISMA flow diagram of study screening and selection PRISMA: Preferred Reporting Items for Systematic Reviews and Meta-Analysis

As shown in Figure 1, the initial database search resulted in 3,970 records. After removing 858 duplicates and excluding 3099 records based on titles and abstracts, thirteen studies were selected for full-text review. Of these, eight studies met the inclusion criteria and were ultimately included in the meta-analysis.

In total, 896 patients underwent TME plus LPLND for treatment of rectal cancer, of whom 470 (52.4%) underwent laparoscopic and 426 (47.6%) open surgery. Males constituted 63% of the study population. The pooled mean age was 57.5 ± 13.15 years in the laparoscopic group and 58.5 ± 12.6 years in the open group [10-17]. The pooled mean body mass index (BMI), based on studies reporting mean ± SD, was 24.3 ± 3.7 kg/m² in the laparoscopic group and 23.8 ± 3.6 kg/m² in the open group [10,11,13,17]. Five of the eight included studies reported the American Society of Anesthesiologists (ASA) as a sample characteristic, with 93% of patients having an ASA score ≤ II [10-14]. The pooled mean follow-up duration was 38.4 ± 17.7 months in the laparoscopic group and 42.1 ± 15.2 months in the open group [11-13,15,17].

**Table 1:** Baseline characteristics of the included studies SD: standard deviation, R-Obs: retrospective observational study, BMI: body mass index, ASA: American Society of Anesthesiologists, NA: not available, L: laparoscopic, O: open * data in median without range or interquartile range, ** data in n (%), *** standard deviation was not reported in the text

Author	Country of origin	Study design	L / O	Male n (%) L / O	Age (years) mean ± SD L / O	BMI (kg/m²) mean ± SD L / O	ASA n (%) L / O	Follow-up (months) mean ± SD L / O
Tang et al., 2022 [10]	China	R-Obs	100 / 100	65 (65) / 62 (62)	57.75 ± 13.75 / 53.0 ± 15.5	24.325 ± 3.875 / 24.65 ± 4.30	I-II: 98 (98) / 100 (100) III: 2 (2) / 0 (0)	39 / 46 *
Lim et al., 2022 [11]	South Korea	R-Obs	126 / 70	73 (57.9) / 39 (55.7)	56.75 ± 12.75 / 57.75 ± 12.75	25.225 ± 4.225 / 24.65 ± 4.30	I-II: 124 (98.4) / 67 (95.7) III-IV: 2 (1.6) / 3 (4.3)	28,9 ± 18,3 / 38,475 ± 17,425
Zhang et al., 2021 [12]	China	R-Obs	39 / 13	16 (41) / 5 (38.5)	55 ± 13.5 / 55.75 ± 9.25	BMI < 24: 17 (43.6) / 8 (61.5) BMI ≥ 24: 22 (56.4) / 5 (38.5) **	II: 26 (66.7) / 10 (76.9) III: 13 (33.3) / 3 (23.1)	58 ± 34.5 both
Yang et al., 2020 [13]	China	R-Obs	30 / 34	19 (63.3) / 21 (61.8)	57.7 ± 12.6 / 61.2 ± 13.3	23.2 ± 2.5 / 23.8 ± 2.7	I-II: 20 (66.7) / 23 (67.6) III-IV: 10 (33.3) / 11 (32.4)	46 ± 23.75 both
Yamaguchi et al., 2017 [14]	Japan	R-Obs	118 / 118	81 (68.6) / 82 (69.5)	57 ± 14 / 61,5 ± 11	BMI < 25: 90 (76.3) / 95 (80.5) BMI ≥ 25: 28 (23.7) / 23 (19.5) **	I-II: 116 (98.3) / 115 (97.5) III: 2 (1.7) / 3 (2.5)	43 / 41.6 *
Nonaka et al., 2017 [15]	Japan	R-Obs	27 / 17	23 (85.2) / 9 (52.9)	64.9 / 62.4 ***	NA	NA	31.5 ± 12 / 48.75 ± 14.25
Mohohashi et al., 2016 [16]	Japan	R-Obs	18 / 61	14 (77.8) / 39 (63.9)	65 ± 8 / 59.75 ± 11.25	22.4 / 22.1*	NA	NA
Matsumoto and Akira, 2016 [17]	Japan	R-Obs	12 / 13	9 (75) / 8 (61.5)	65.6 ± 8.7 / 68.3 ± 6.5	21.0 ± 1.8 / 21.4 ± 3.8	NA	24.4 ± 8.3 / 31.5 ± 14.7

The pooled mean distance from the anal verge was 5.09 ± 2.78 cm in the laparoscopic group and 5.05 ± 2.67 cm in the open group, with similar pooled mean tumor sizes of 4.38 ± 1.52 cm and 4.30 ± 1.85 cm, respectively [10-14,17]. Neoadjuvant therapy was administered to 46.8% of patients undergoing laparoscopic surgery and 35.5% of those undergoing open surgery, while adjuvant therapy was administered to 61% and 59.7% of patients, respectively [10-15]. Previous abdominal surgery was documented in 18% of laparoscopic cases and 19% of open cases [10,11,13,14]. The distribution of pathological TNM stage was comparable between groups, with stage < III in 46.5% of laparoscopic cases and 46.3% of open cases, and stage ≥ III in 53.5% and 53.7%, respectively [10,14-17].

Diverting stomas were more frequently created in the laparoscopic group (78.3% vs 69.7%) [10,11,13,14]. Unilateral lateral pelvic node dissection was performed in 57% of laparoscopic cases and 46.8% of open cases, whereas bilateral dissection occurred in 43% and 53.2%, respectively [10-15]. Regarding the type of resection, low anterior resection was performed in 63% of laparoscopic cases and 53.5% of open cases; abdominoperineal resection in 26% and 29%; and intersphincteric resection in 8% and 13%, respectively [10-14,16,17]. Additional study characteristics are detailed in Tables 1-2.

**Table 2:** Surgical and pathological characteristics of the included studies SD: standard deviation, AV: anal verge, Neoadj: neoadjuvant treatment, Adjuv: adjuvant treatment, Pre-abd surgery: previous abdominal surgery, LPNM: lateral pelvic nodes metastasis, LPND: lateral pelvic nodes dissection, L: laparoscopic, O: open * Patients with anastomosis → 81/118 in the lap group had an anastomosis, and 77/118 in the open group

Author	Distance from AV (cm) mean ± SD L / O	Tumor size (cm) mean ± SD L / O	Neoadj n (%) L / O	Adjuv n (%) L / O	Pre-abd surgery n (%) L / O	pTNM stage n (%) L / O	LPNM n (%) L / O	Type of operation n (%) L / O	Diverting ostomy n (%) L / O	Extent of LPND n (%) L / O
Tang et al., 2022 [10]	4.25 ± 2 / 4.0 ± 2	NA	21 (21) / 15 (15)	NA	15 (15) / 20 (20)	I: 14 (14) / 17 (17), II: 35 (35) / 36 (36), III: 51 (51) / 47 (47)	24 (24) / 19 (19)	Low anterior: 42 (42) / 48 (48), abdominoperineal: 52 (52) / 48 (48), Hartmann: 6 (6) / 4 (4)	66 (66) / 67 (67)	Unilateral: 38 (38) / 32 (32), bilateral: 62 (62) / 68 (68)
Lim et al., 2022 [11]	6.25 ± 3.75 / 6.25 ± 3.75	NA	108 (85.7) / 53 (75.7)	NA	38 (30.2) / 21 (30)	NA	32 (25.4) / 25 (35.7)	Low anterior: 93 (73.8) / 43 (61.4), abdominoperineal: 11 (8.7) / 13 (18.6), intersphincteric: 22 (17.5) / 14 (20)	119 (94.4) / 63 (90)	Unilateral: 103 (81.7) / 48 (68.6), bilateral: 23 (18.3) / 22 (31.4)
Zhang et al., 2021 [12]	5.25 ± 2.25 / 5.25 ± 2.25	4.125 ± 1.625 / 4.5 ± 1.5	23 (59) / 6 (42.6)	NA	NA	NA	11 (28.2) / 7 (53.8)	Low anterior: 27 (69.2) / 7 (53.8), abdominoperineal: 7 (17.9) / 6 (46.2), intersphincteric: 4 (10.3) / 0 (0), Hartmann: 1 (2.6) / 0 (0)	NA	Unilateral: 29 (74.4) / 9 (69.2), bilateral: 10 (25.6) / 4 (30.8)
Yang et al., 2020 [13]	5.3 ± 2.1 / 5.9 ± 1.7	4.8 ± 1.5 / 4.2 ± 2.1	21 (70) / 22 (64.7)	24 (80) / 25 (73.5)	2 (6.7) / 4 (11.8)	NA	11 (36.7) / 10 (29.4)	Low anterior: 21 (70) / 27 (79.4), abdominoperineal: 3 (10) / 2 (5.9), intersphincteric: 6 (20) / 5 (14.7)	17 (56.7) / 22 (64.7)	Unilateral: 27 (90) / 29 (85.3), bilateral: 3 (10) / 5 (14.7)
Yamaguchi et al., 2017 [14]	4.5 ± 2.5 / 5 ± 2.75	NA	28 (23.7) / 28 (23.7)	72 (61.5) / 65 (55.6)	12 (10.2) / 16 (13.6)	0-II: 54 (45.8) / 46 (39.0), III: 64 (54.2) / 72 (61.0)	24 (20.3) / 17 (14.4)	Low anterior: 81 (68.6) / 77 (65.3), abdominoperineal: 36 (30.5) / 32 (27.1), Hartmann: 0 (0.0) / 4 (3.4), pelvic exenteration: 1 (0.8) / 5 (4.2)	62 (76.5) / 44 (57.9) *	Unilateral:36 (30.5) / 36 (30.5), bilateral: 82 (69.5) / 82 (69.5)
Nonaka et al., 2017 [15]	NA	NA	5 (18.5) / 1 (5.9)	11 (40.7) / 11 (64.7)	NA	I: 7 (25.9) / 2 (11.8), II: 5 (18.5) / 3 (17.6), III: 15 (55.6) / 12 (70.6)	NA	Sphincter preserving: 21 (77.3) / 9 (52.6)	NA	Unilateral: 18 (66.7) / 11 (64.7), bilateral: 9 (33.3) / 6 (35.3)
Mohohashi et al., 2016 [16]	NA	NA	NA	NA	NA	0-II: 8 (44.4) / 34 (55.7), ≥ III: 10 (55.6) / 27 (44.3))	8 (44.4) / 25 (41)	Low anterior: 8 (44.4) / 15 (24.6), abdominoperineal: 5 (27.8) / 19 (31.1), intersphincteric: 3 (16.7) / 23 (37.7), Hartmann: 2 (11.1) / 4 (6.6)	NA	NA
Matsumoto and Akira, 2016 [17]	4.75 ± 1.42 / 4.77 ± 1.59	4.13 ± 1.19 / 4.35 ± 1.35	NA	NA	NA	0-II: 5 (41.7) / 5 (38.5), ≥ III: 7 (58.3) / 8 (61.5)	3 (25) / 4 (30.8)	Low anterior: 8 (66.7) / 2 (15.4), abdominoperineal: 3 (25) / 0 (0), intersphincteric: 1 (8.3) / 11 (84.6)	NA	NA

Pooled Analyses of All Studies

**Figure 2:**
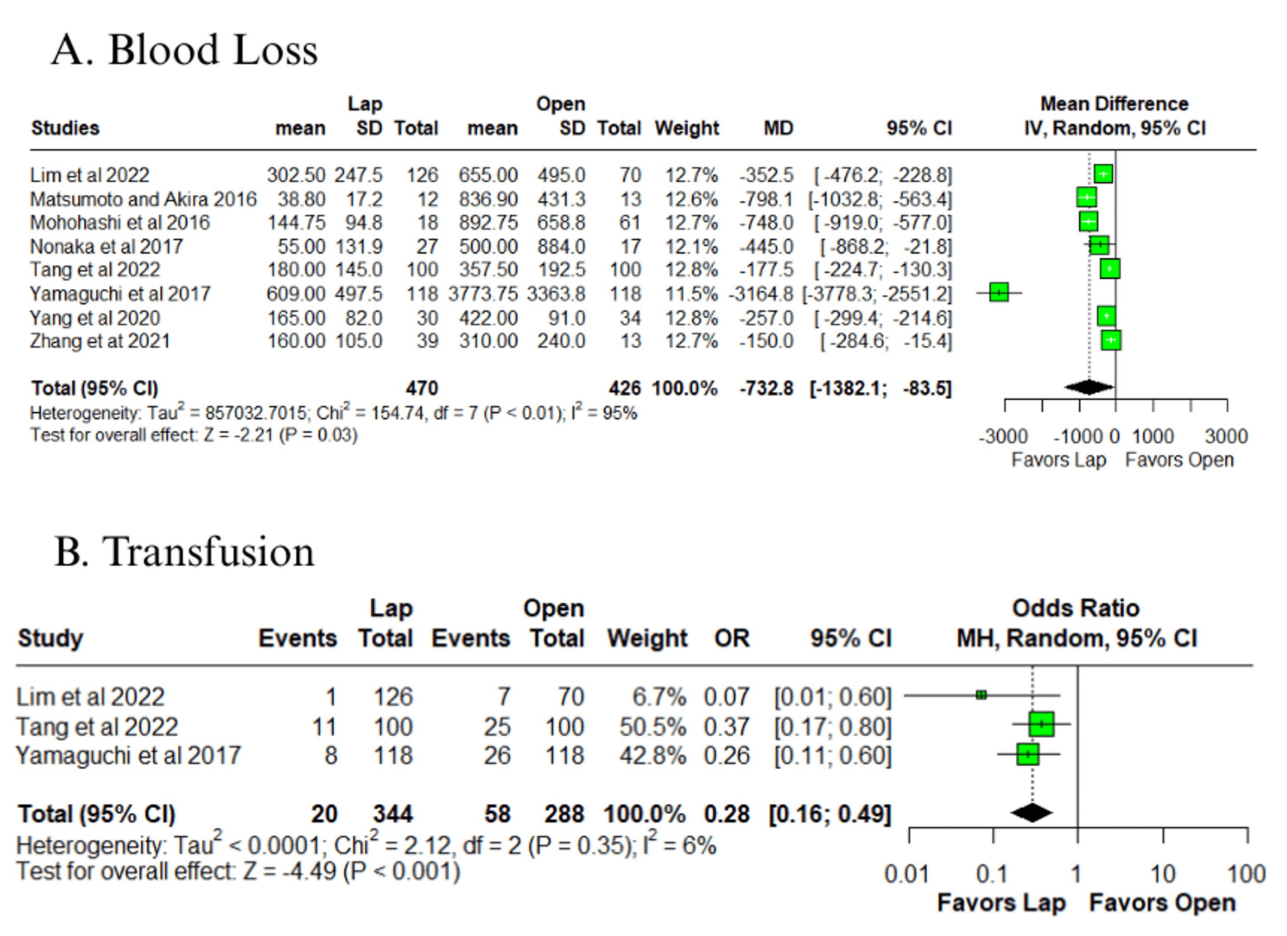
Forest plot of comparison laparoscopic vs open: (A) blood loss and (B) transfusion Tang et al., 2022 [10], Lim et al., 2022 [11], Zhang et al., 2021 [12], Yang et al., 2020 [13], Yamaguchi et al., 2017 [14], Nonaka et al., 2017 [15], Mohohashi et al., 2016 [16], and Matsumoto and Akira, 2017 [17] Lap: laparoscopic, SD: standard deviation, MD: mean difference, CI: confidence interval, IV: inverse variance, OR: odds ratio, MH: Mantel–Haenszel

**Figure 3:**
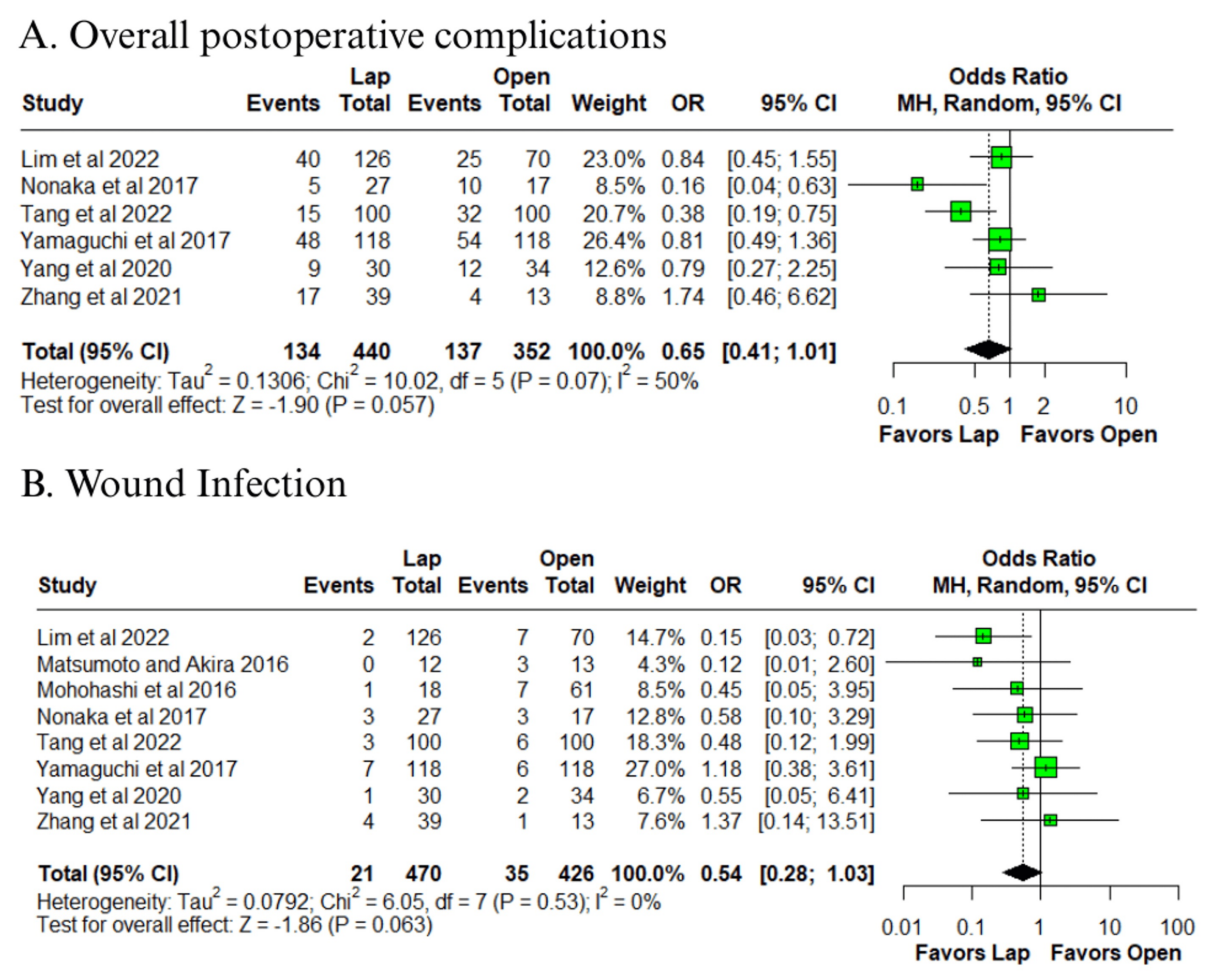
Forest plot of comparison laparoscopic vs open: (A) overall postoperative complications and (B) wound infection Tang et al., 2022 [10], Lim et al., 2022 [11], Zhang et al., 2021 [12], Yang et al., 2020 [13], Yamaguchi et al., 2017 [14], Nonaka et al., 2017 [15], Mohohashi et al., 2016 [16], and Matsumoto and Akira, 2017 [17] Lap: laparoscopic, SD: standard deviation, MD: mean difference, CI: confidence interval, IV: inverse variance, OR: odds ratio, MH: Mantel–Haenszel

In the pooled analysis, the laparoscopic group showed lower intraoperative blood loss (MD = −732.8 ml; 95% CI: −1328.1 to −83.5; p = 0.03; I² = 95%) (Figure 2A) [10-17], although this was associated with very high heterogeneity. Consistently, the laparoscopic group also had a lower need for blood transfusion (OR = 0.28; 95% CI: 0.16 to 0.49; p < 0.0001; I² = 6%) (Figure 2B) [10,11,14], with low heterogeneity. Trends favoring the laparoscopic group were observed for overall postoperative complications (OR = 0.65; 95% CI: 0.41 to 1.01; p = 0.057; I² = 50%) (Figure 3A) [10-15] and wound infection (OR = 0.54; 95% CI: 0.28 to 1.03; p = 0.063; I² = 0%) (Figure 3B) [10-17].

**Figure 4:**
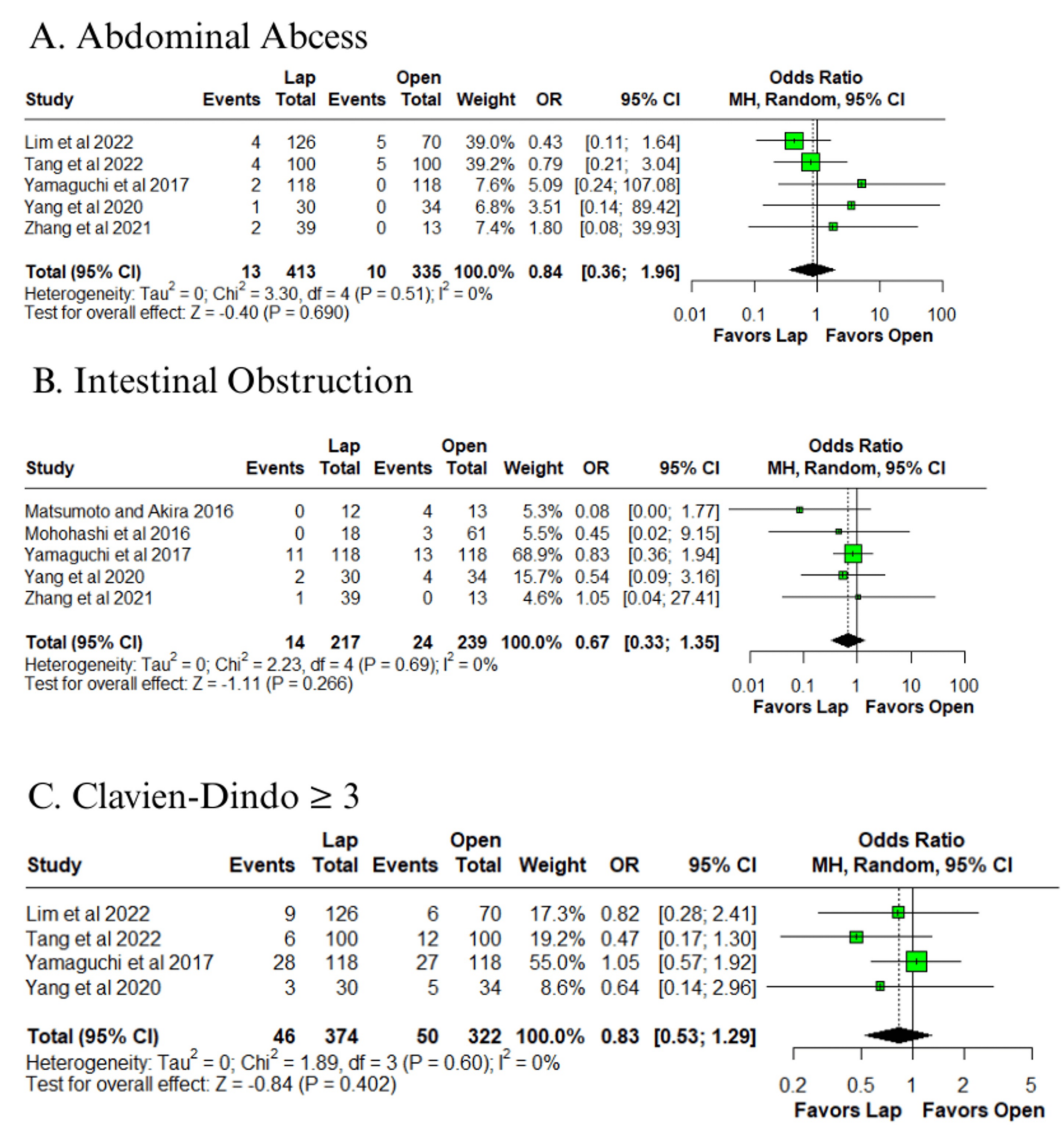
Forest plot of comparison laparoscopic vs open: (A) abdominal abscess, (B) intestinal obstruction, and (C) Clavien-Dindo ≥ 3 Tang et al., 2022 [10], Lim et al., 2022 [11], Zhang et al., 2021 [12], Yang et al., 2020 [13], Yamaguchi et al., 2017 [14], Mohohashi et al., 2016 [16], and Matsumoto and Akira, 2017 [17] Lap: laparoscopic, SD: standard deviation, MD: mean difference, CI: confidence interval, IV: inverse variance, OR: odds ratio, MH: Mantel–Haenszel

**Figure 5:**
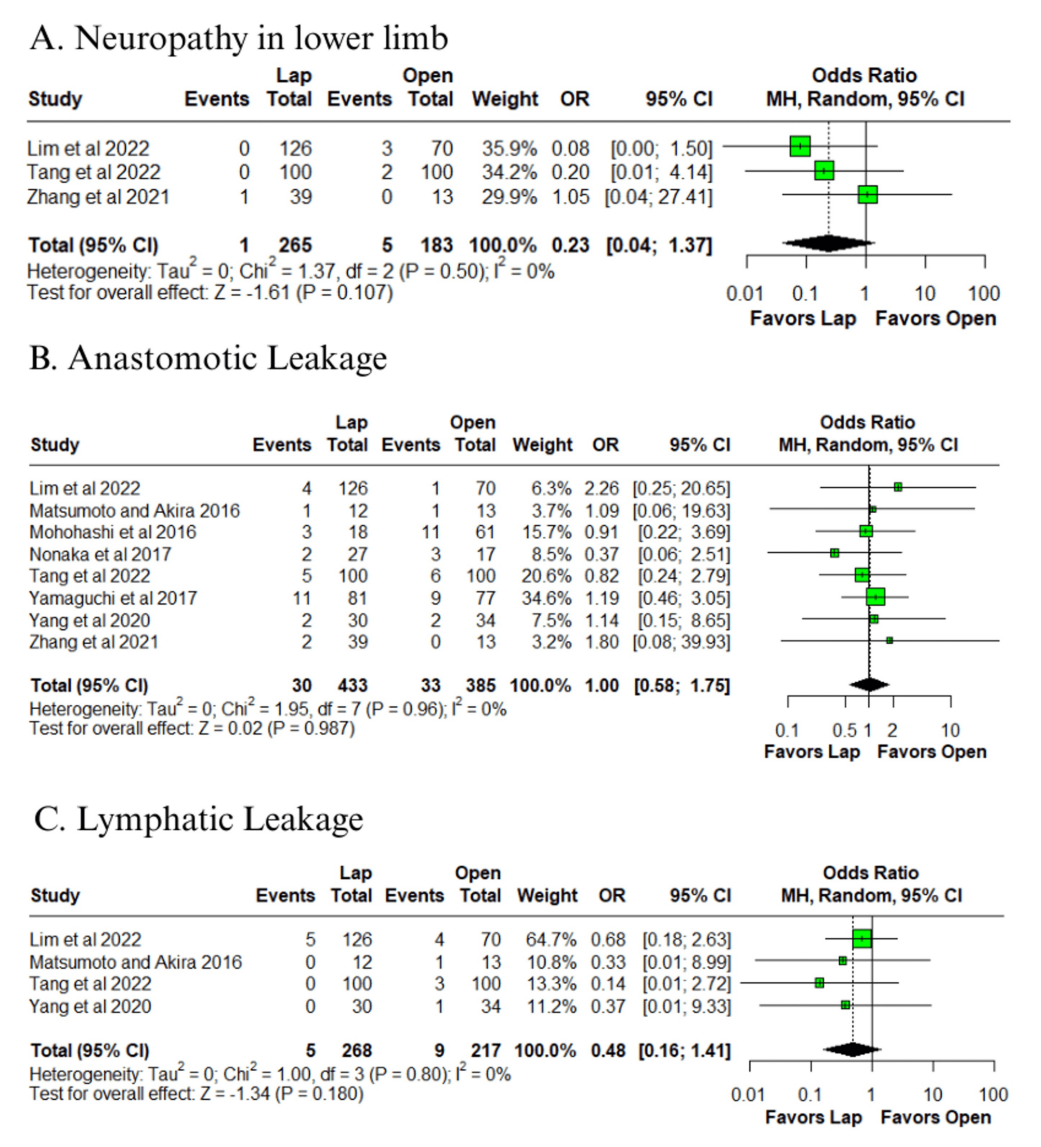
Forest plot of comparison laparoscopic vs open: (A) neuropathy in lower limb, (B) anastomotic leakage, and (C) lymphatic leakage Tang et al., 2022 [10], Lim et al., 2022 [11], Zhang et al., 2021 [12], Yang et al., 2020 [13], Yamaguchi et al., 2017 [14], Nonaka et al., 2017 [15], Mohohashi et al., 2016 [16], and Matsumoto and Akira, 2017 [17] Lap: laparoscopic, SD: standard deviation, MD: mean difference, CI: confidence interval, IV: inverse variance, OR: odds ratio, MH: Mantel–Haenszel

Among the outcomes with low heterogeneity (I² ≤ 25%), no statistically significant differences were observed between groups for abdominal abscess (OR = 0.84; 95% CI: 0.36 to 1.96; p = 0.690; I² = 0%) (Figure 4A) [10-14], intestinal obstruction (OR = 0.67; 95% CI: 0.33 to 1.35; p = 0.266; I² = 0%) (Figure 4B) [12-14,16,17], Clavien-Dindo complications grade ≥ 3 (OR = 0.83; 95% CI: 0.53 to 1.29; p = 0.402; I² = 0%) (Figure 4C) [10,11,13,14], lower limb neuropathy (OR = 0.23; 95% CI: 0.04 to 1.37; p = 0.107; I² = 0%) (Figure 5A) [10-12], anastomotic leakage (OR = 1.00; 95% CI: 0.58 to 1.75; p = 0.987; I² = 0%) (Figure 5B) [10-17], and lymphatic leakage (OR = 0.48; 95% CI: 0.16 to 1.41; p = 0.180; I² = 0%) (Figure 5C) [10,11,13,17].

**Figure 6:**
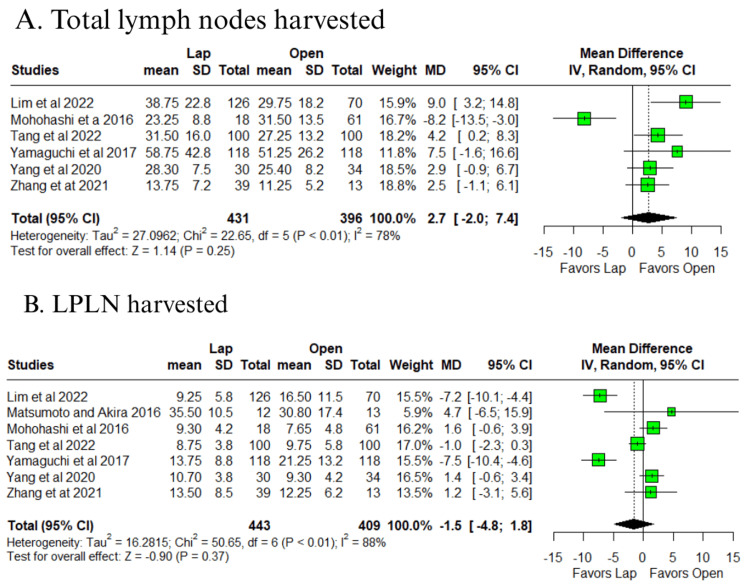
Forest plot of comparison laparoscopic vs open: (A) total lymph nodes harvested and (B) LPLN harvested Tang et al., 2022 [10], Lim et al., 2022 [11], Zhang et al., 2021 [12], Yang et al., 2020 [13], Yamaguchi et al., 2017 [14], Mohohashi et al., 2016 [16], and Matsumoto and Akira, 2017 [17] LPLN: lateral pelvic lymph node, Lap: laparoscopic, SD: standard deviation, MD: mean difference, CI: confidence interval, IV: inverse variance, OR: odds ratio, MH: Mantel–Haenszel

**Figure 7:**
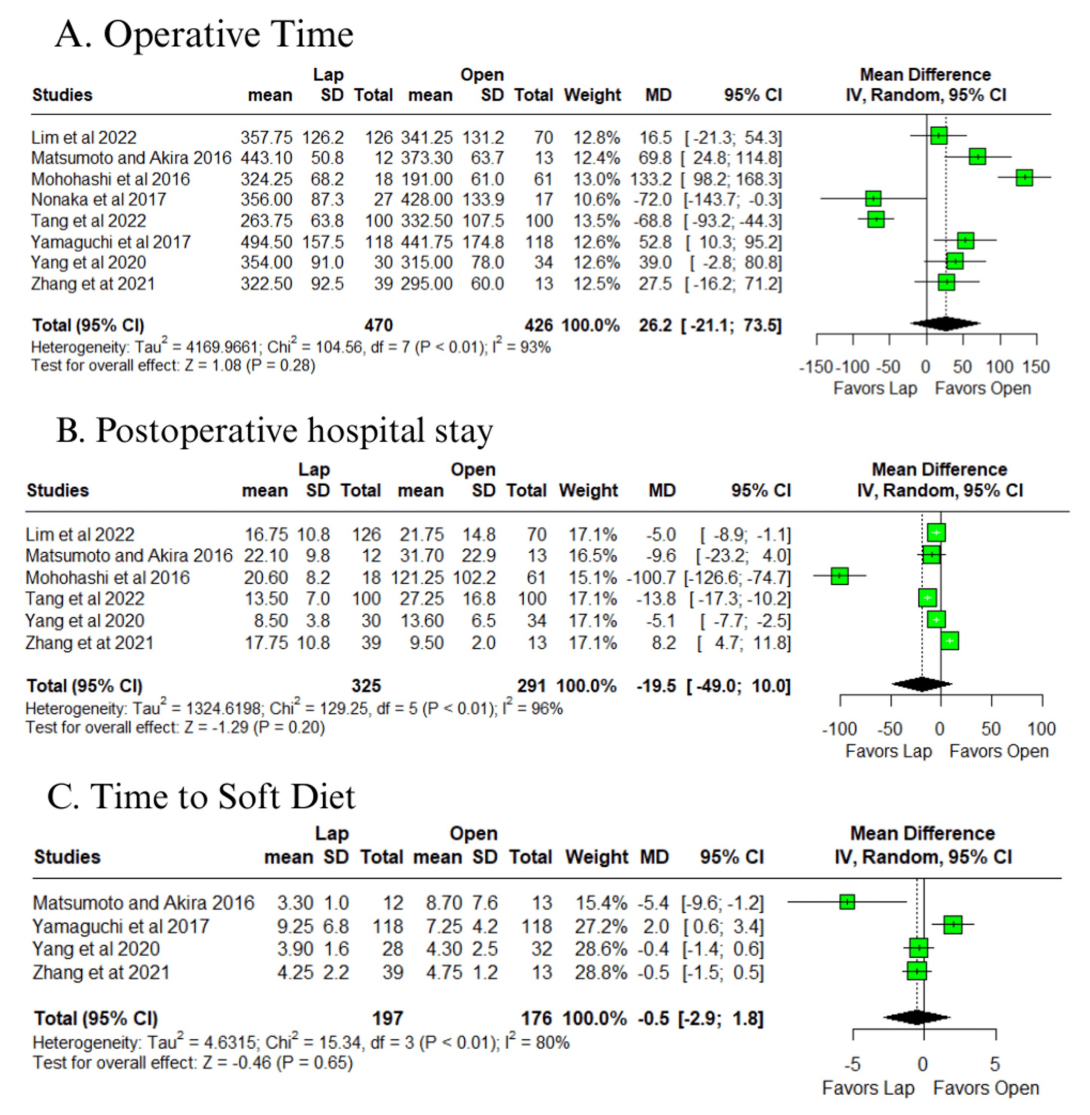
Forest plot of comparison laparoscopic vs open: (A) operative time, (B) postoperative hospital stay, and (C) time to soft diet Tang et al., 2022 [10], Lim et al., 2022 [11], Zhang et al., 2021 [12], Yang et al., 2020 [13], Yamaguchi et al., 2017 [14], Mohohashi et al., 2016 [16], and Matsumoto and Akira, 2017 [17] Lap: laparoscopic, SD: standard deviation, MD: mean difference, CI: confidence interval, IV: inverse variance, OR: odds ratio, MH: Mantel–Haenszel

In outcomes with high or very high heterogeneity (I² > 50%), no significant differences were identified for total lymph nodes harvested (MD = 2.7; 95% CI: −2.0 to 7.4; p = 0.25; I² = 78%) (Figure 6A) [10-14,16], LPLN harvested (MD = −1.5; 95% CI: −4.8 to 1.8; p = 0.37; I² = 88%) (Figure 6B) [10-14,16,17], operative time (MD = 26.2 minutes; 95% CI: −21.1 to 73.5; p = 0.28; I² = 93%) (Figure 7A) [10-17], postoperative hospital stay (MD = −19.5 days; 95% CI: −49 to 10; p = 0.20; I² = 96%) (Figure 7B) [10-13,16,17], and time to soft diet (MD = −0.5 days; 95% CI: −2.9 to 1.8; p = 0.65; I² = 80%) (Figure 7C) [12-14,17].

Sensitivity Analyses

**Figure 8:**
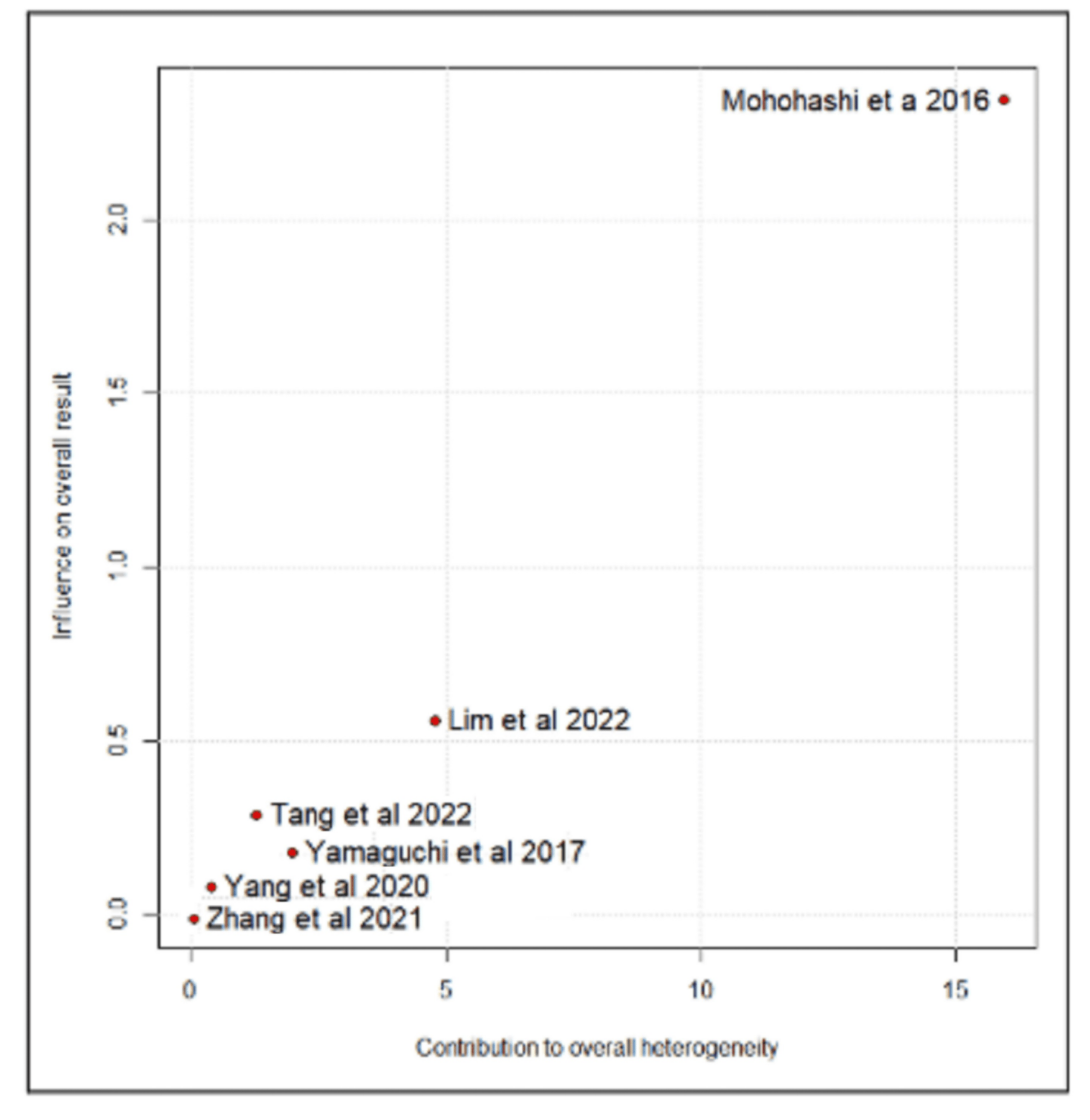
Baujat plot for total lymph nodes harvested Tang et al., 2022 [10], Lim et al., 2022 [11], Zhang et al., 2021 [12], Yang et al., 2020 [13], Yamaguchi et al., 2017 [14] and Mohohashi et al., 2016 [16]

**Figure 9:**
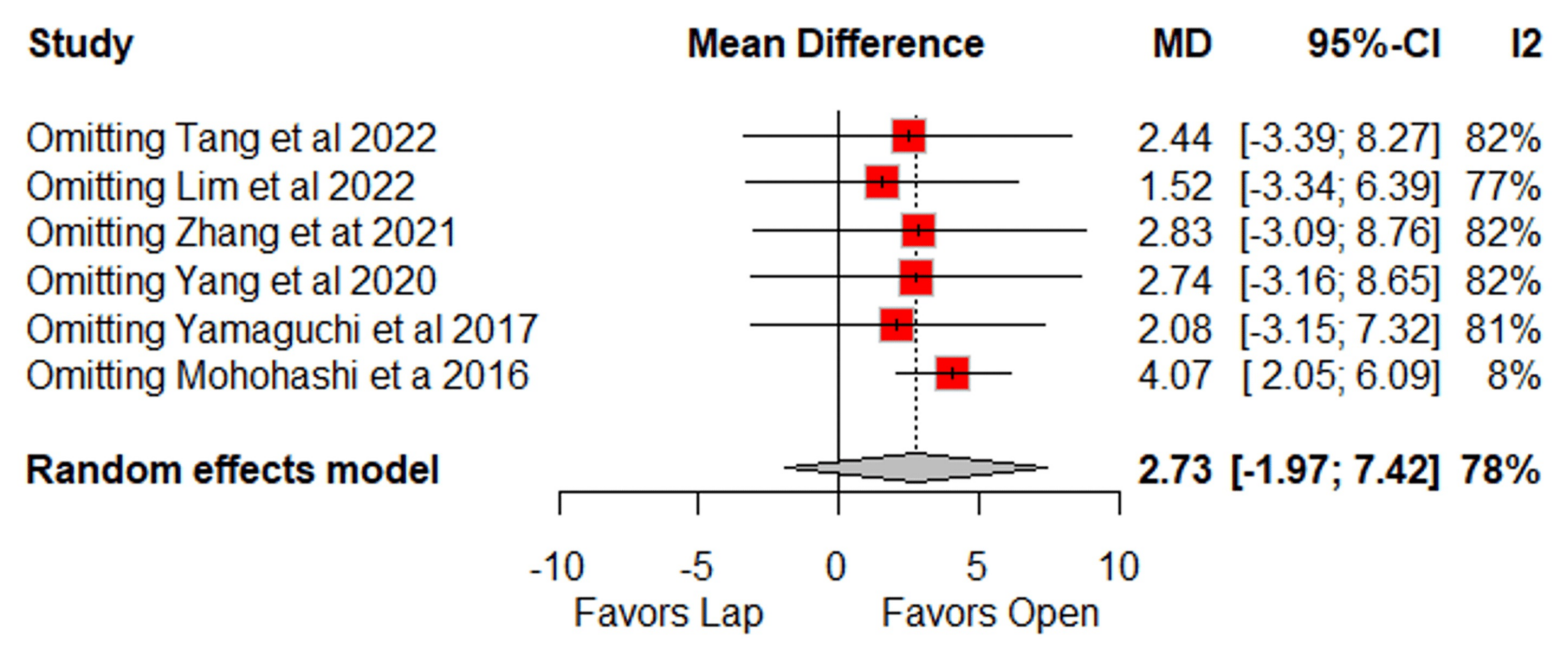
Leave-one-out sensitivity for total lymph nodes harvested Tang et al., 2022 [10], Lim et al., 2022 [11], Zhang et al., 2021 [12], Yang et al., 2020 [13], Yamaguchi et al., 2017 [14] and Mohohashi et al., 2016 [16] MD: mean difference, CI: confidence interval

The Baujat plot analysis identified the studies that contributed most to heterogeneity. Among the total lymph nodes harvested, Mohohashi et al. were the main contributors. After its removal in the leave-one-out analysis, there was a significant difference in the number of lymph nodes harvested, favoring the open surgery group (MD = 4.07; 95% CI: 2.05 to 6.09; I² = 8%) (Figures 8-9) [10-14,16].

**Figure 10:**
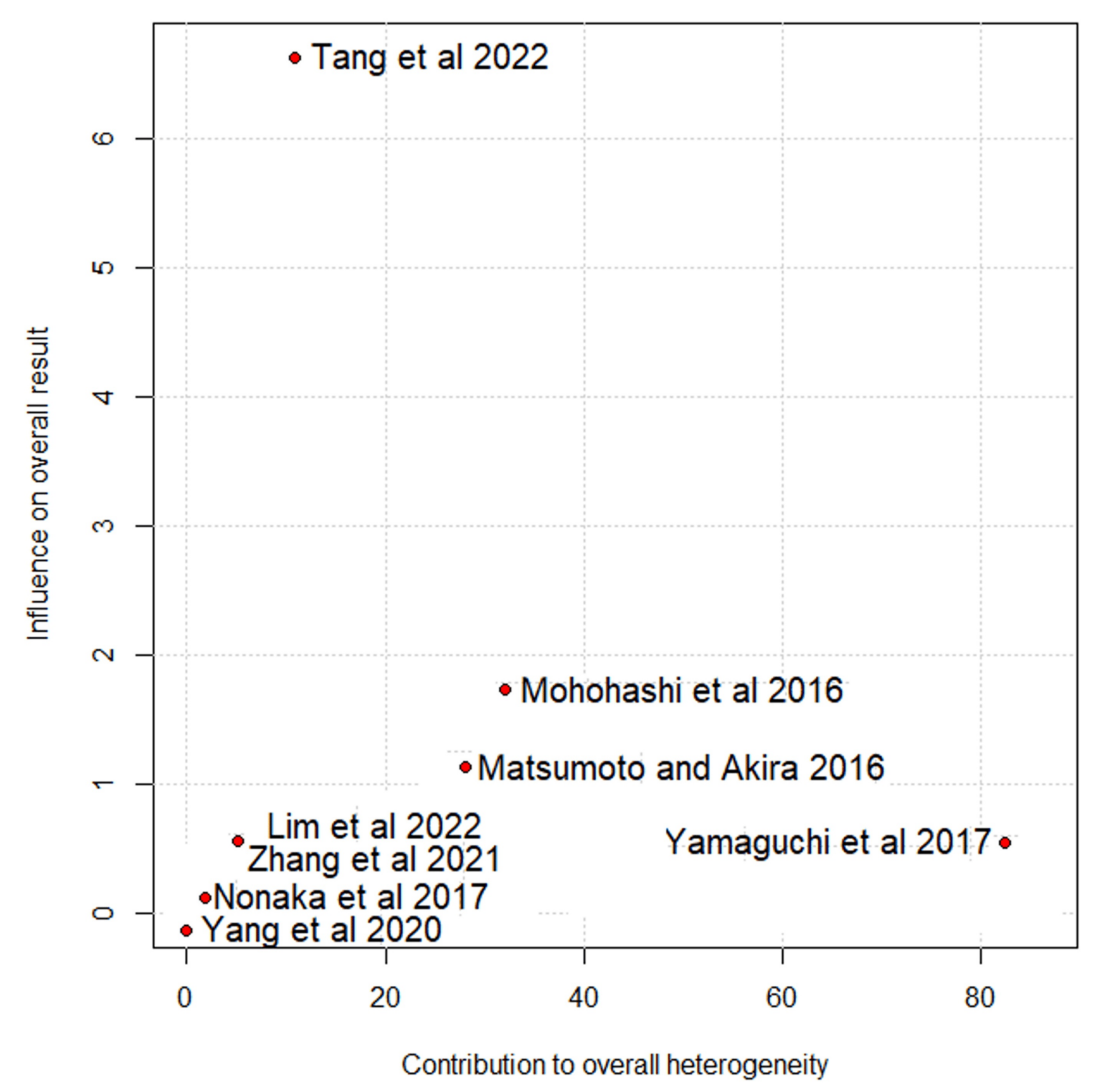
Baujat plot for blood loss Tang et al., 2022 [10], Lim et al., 2022 [11], Zhang et al., 2021 [12], Yang et al., 2020 [13], Yamaguchi et al., 2017 [14], Nonaka et al., 2017 [15], Mohohashi et al., 2016 [16], and Matsumoto and Akira, 2017 [17]

**Figure 11:**
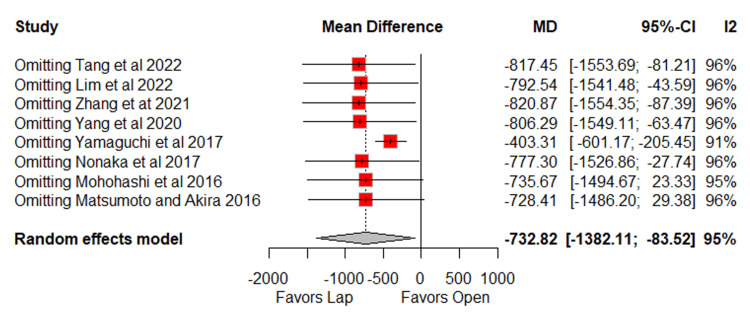
Leave-one-out sensitivity for blood loss Tang et al., 2022 [10], Lim et al., 2022 [11], Zhang et al., 2021 [12], Yang et al., 2020 [13], Yamaguchi et al., 2017 [14], Nonaka et al., 2017 [15], Mohohashi et al., 2016 [16], and Matsumoto and Akira, 2017 [17] MD: mean difference, CI: confidence interval

Blood loss differed significantly between groups, with high heterogeneity mainly attributable to Yamaguchi et al. After excluding this study from the sensitivity analysis, the effect remained statistically significant, and heterogeneity persisted at a high level (MD = −403.31; 95% CI: −601.17 to −205.45; I² = 91%) (Figures 10-11) [10-17].

**Figure 12:**
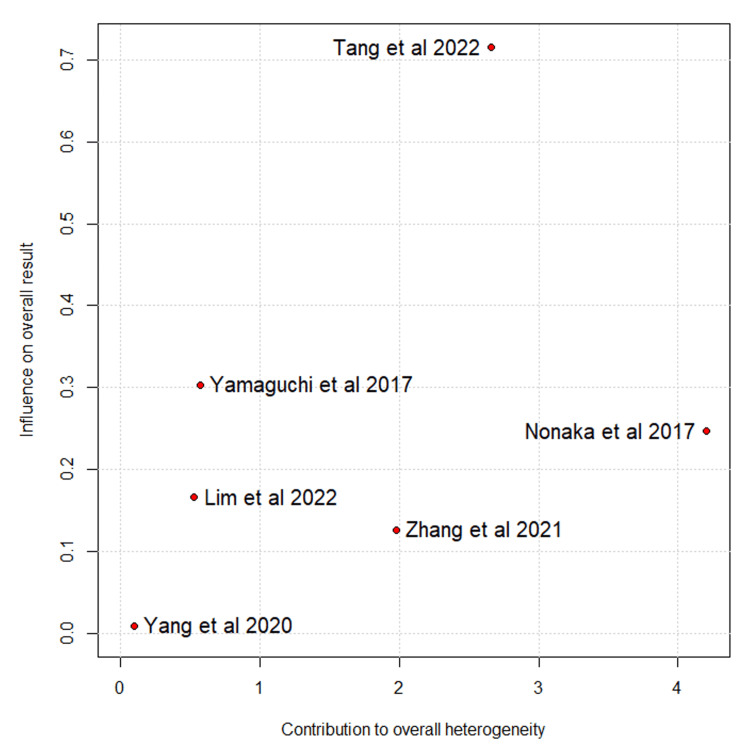
Baujat plot for overall postoperative complications Tang et al., 2022 [10], Lim et al., 2022 [11], Zhang et al., 2021 [12], Yang et al., 2020 [13], Yamaguchi et al., 2017 [14] and Nonaka et al., 2017 [15]

**Figure 13:**
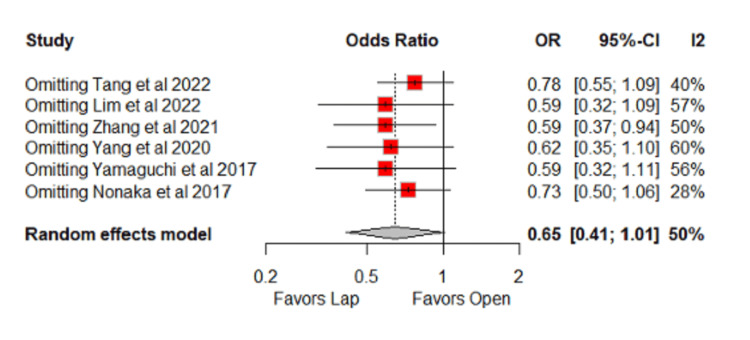
Leave-one-out sensitivity for overall postoperative complications Tang et al., 2022 [10], Lim et al., 2022 [11], Zhang et al., 2021 [12], Yang et al., 2020 [13], Yamaguchi et al., 2017 [14] and Nonaka et al., 2017 [15] OR: odds ratio, CI: confidence interval

For overall postoperative complications, no significant difference was observed, with moderate heterogeneity (OR = 0.65; 95% CI: 0.41 to 1.01; p = 0.057; I² = 50%), mainly attributable to Nonaka et al. After its exclusion from the leave-one-out sensitivity analysis, the results remained non-significant, and heterogeneity persisted at a moderate level (OR = 0.73; 95% CI: 0.50 to 1.06; I² = 28%) (Figures 12-13) [10-15].

Quality Assessment

**Figure 14:**
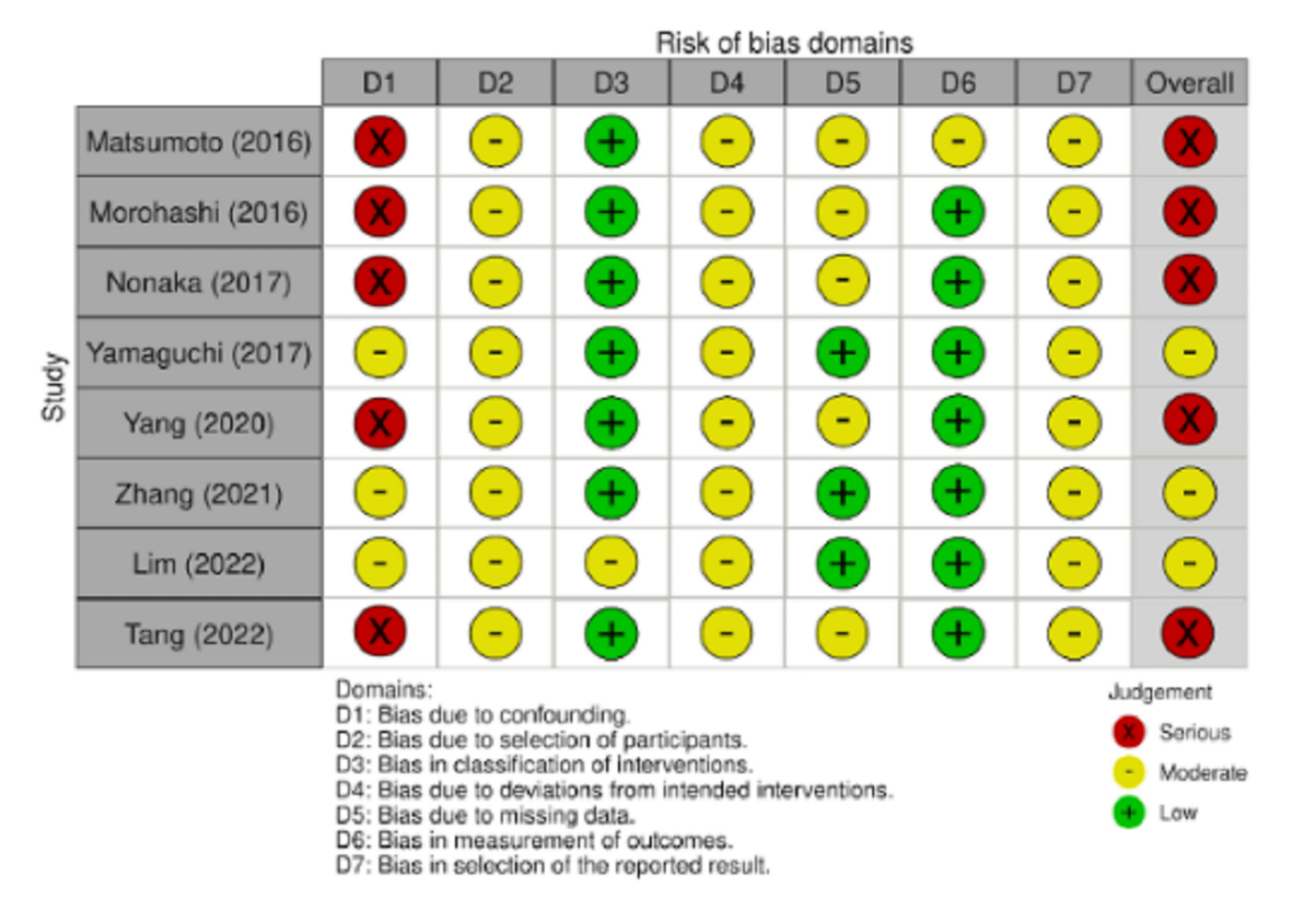
Critical appraisal of cohort studies using the Cochrane Collaboration's tool for assessing risk of bias in non-randomized studies of interventions (ROBINS-I) Tang et al., 2022 [10], Lim et al., 2022 [11], Zhang et al., 2021 [12], Yang et al., 2020 [13], Yamaguchi et al., 2017 [14], Nonaka et al., 2017 [15], Mohohashi et al., 2016 [16], and Matsumoto and Akira, 2017 [17]

Overall ROBINS-I ratings are shown in Figure 14. Tang et al., Yang et al., Nonaka et al., Morohashi et al., and Matsumoto et al. were judged at serious risk of bias, whereas Lim et al., Zhang et al., and Yamaguchi et al. were rated as moderate. The main driver of the overall serious ratings was confounding bias. All studies were rated as moderate for participant selection, selection of reported results, and deviations from intended interventions. The classification of interventions was judged to be moderate only in Lim et al. Missing data were judged to be moderate in Tang et al., Yang et al., Nonaka et al., Morohashi et al., and Matsumoto et al. Measurement of outcomes was judged to be moderate only in Matsumoto et al. [10-17].

Discussion

The present study demonstrated statistically significant reductions in intraoperative blood loss and transfusion requirements, along with a positive trend toward fewer overall postoperative complications and wound infections. These findings are consistent with those reported by Watanabe et al., who also observed lower bleeding rates and fewer postoperative complications, and with those reported by Ouyang et al., who confirmed an association with reduced intraoperative bleeding [18,19].

A plausible explanation for the lower blood loss observed with laparoscopy is the magnified, high-definition view in the narrow, deep pelvis, which can facilitate more precise dissection, clearer identification of vascular structures, and earlier, more controlled hemostasis. Additionally, surgeons may be less tolerant of even minor bleeding during laparoscopy because blood quickly degrades visualization, and uncontrolled hemorrhage is a common reason for conversion to open surgery, a factor that encourages meticulous hemostatic technique throughout the procedure [20-21].

The reduced need for transfusion is clinically relevant because allogeneic blood transfusion has been associated with poorer postoperative outcomes, including higher rates of infectious complications. These effects are commonly attributed, at least in part, to transfusion-related immunomodulation and broader immune-inflammatory responses triggered by transfused blood products. From a perioperative safety perspective, minimizing transfusion also reduces exposure to recognized adverse events, including transfusion reactions and cardiopulmonary complications such as transfusion-associated circulatory overload and transfusion-related acute lung injury, which can delay recovery [22-24].

According to the literature, laparoscopic LLND has been associated with a lower risk of postoperative complications compared with the open approach, as reported by Watanabe et al. In our pooled analysis, overall complications also favored laparoscopy; however, the difference did not reach statistical significance, despite the observed trend. Operative time is strongly influenced by technical complexity, case selection, and surgeon experience, so differences across centers and learning curves may dilute pooled effects. In our meta-analysis, most included studies reported shorter operative times with the open approach, which is consistent with the broader literature [18]. In contrast, Tang et al. and Nonaka et al. deviated from this pattern, which may reflect their setting in tertiary, high-volume centers with specialized laparoscopic expertise [10,15,18].

Some limitations remain, as most of the available evidence derives from retrospective, non-randomized studies, with a generally high risk of bias based on the Risk of Bias (RoB) assessment, relatively small and heterogeneous samples, and follow-up periods shorter than five years. These factors warrant caution in interpreting the findings and reinforce the need for larger, well-designed randomized controlled trials with long-term outcomes to validate the observed advantages.

## Conclusions

This systematic review and meta-analysis suggest that the laparoscopic approach to LPLND following TME for advanced rectal cancer is a feasible and safe alternative to the open approach. Laparoscopic LPLND was associated with significantly lower intraoperative blood loss and a reduced need for blood transfusion while also showing favorable trends toward fewer overall postoperative complications and wound infections.

These findings support the growing role of minimally invasive surgery in the management of locally advanced rectal cancer requiring LPLND, particularly in centers with appropriate technical expertise. However, the interpretation of these results should remain cautious, as the available evidence is based exclusively on retrospective non-randomized studies, many of which were judged to have moderate or serious risk of bias, with substantial heterogeneity across several pooled analyses and relatively limited follow-up in part of the included literature.
